# Sonophotocatalytic degradation of sulfamethoxazole using lanthanum ferrite perovskite oxide anchored on an ultrasonically exfoliated porous graphitic carbon nitride nanosheet

**DOI:** 10.1039/d4ra03096f

**Published:** 2024-07-12

**Authors:** Ajibola A. Bayode, Andrea Osti, Antonella Glisenti

**Affiliations:** a Department of Chemical Sciences, Faculty of Natural Sciences, Redeemer's University P.M.B. 230 Ede 232101 Nigeria bayodea@run.edu.ng ajibolabay7@gmail.com; b Department of Chemical Sciences, University of Padova Via F. Marzolo, 1 35131 Padua Italy

## Abstract

The lanthanum ferrite perovskite (La_0.8_FO) was synthesized using a citric combustion route and then modified with a porous graphitic nitride nanosheet *via* the wet impregnation-assisted ultrasonic method to produce La_0_._8_FO@PgNS. Various techniques such as Fourier transform infrared spectroscopy (FTIR), X-ray diffraction (XRD), scanning electron microscopy (SEM), energy dispersive X-ray (EDX) spectroscopy, X-ray photoelectron spectroscopy (XPS), ultraviolet diffuse reflectance spectroscopy (UV-DRS), and Tauc plot analysis were employed to confirm the functional moieties, crystallinity, phase change, morphology, composition, and bandgap of La._0.8_FO and La_0_._8_FO@PgNS. La_0.8_FO and La_0_._8_FO@PgNS were used for the sonophotocatalytic oxidative degradation of sulfamethoxazole (SMX) under low energy and ultrasound wave frequency in the presence of visible light. La_0.8_FO and La_0_._8_FO@PgNS exhibited a sonophotocatalytic degradation capacity of 52.06 and 99.60%, respectively. Furthermore, the rate constant at the optimum condition of pH 7 and 5 mg L^−1^ concentration was 0.01343 and 0.01494 min^−1^ for La_0.8_FO and La_0_._8_FO@PgNS, respectively. The integration of sonolysis and photocatalysis in the remediation process of SMX resulted in a synergy of 2.5-fold. Ultrasonic waves and hydroxyl and superoxide radicals are the main species governing the degradation process while La_0_._8_FO@PgNS was stable over 8 cycles, proving to be a sustainable material for environmental remediation.

## Abstract 2

Lanthanum ferrite, anchored on graphitic carbon nitride nanosheets, was synthesized and can degrade sulfamethoxazole. The degradation occurred through a sono-photocatalytic process in water. The material is reusable for up to eight cycles.

## Graphical abstract

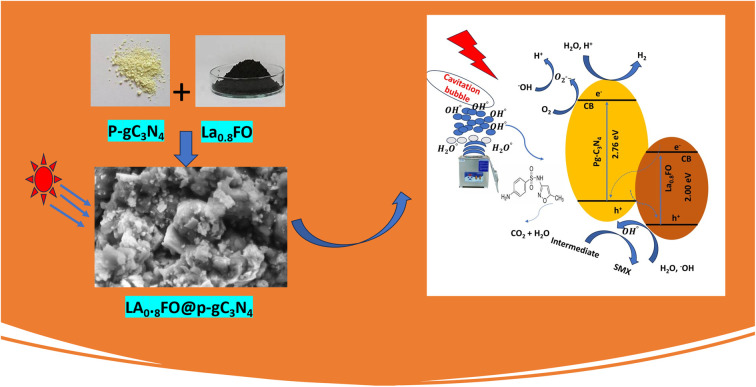

## 1 Introduction

The rapid industrialization and globalization of recent times have resulted in a range of environmental issues, including the presence of pharmaceutical and personal care product (PPCP) residue in aquatic ecosystems.^1,2^ PPCPs are a distinct class of contaminants that have become a global concern due to their adverse impact on the environment and human health. This is a result of the widespread use of PPCPs by people, which has led to their accumulation in various water sources.^3–5^

Antibiotics, particularly sulfamethoxazole (SMX), are a dominant class of pharmaceuticals found in the environment owing to their extensive use and abuse by humans, which has made them a public concern.^6^ Although SMX is highly effective in treating bacterial infections, its excessive or long-term intake can damage the kidneys and reduce body immunity, leading to antibiotics resistance–a major problem the world is currently fighting. SMX residues in surface and ground waters can be harmful to both fauna and flora, making it necessary to find an effective and economical technology for its removal as the current water treatment system is not equipped to handle this problem.^7,8^

Various methods have been employed to eliminate SMX from water, including filtration,^9^ membrane filtration,^10^ adsorption,^11^ electrochemical catalysis,^12^ sonolysis,^13^ coagulation,^14^ biodegradation,^15^ photocatalysis,^16^ ozonation,^17^ and advanced oxidation processes (AOPs).^18,19^ Recently, AOPs such as photocatalysis and sonocatalysis have been receiving much attention owing to their advantage of being able to convert organic water pollutants to low molecular weight substances and the fact that they have proven to be more efficient in eliminating pollutants due to their synergistic effect.

The sonophotocatalytic process is a type of AOP that can degrade multiple pollutants in water. Sonolysis is based on the cavitation phenomenon, producing microbubbles that form transient microreactors with high temperatures and pressures, dissociating water molecules to form ˙OH radicals.^20^ When stimulated by light or ultrasonic irradiation, semiconductors create electron and hole (e^−^/h^+^) pairs in their conduction and valence bands, respectively, and subsequently form reactive oxygen species (ROS) that effectively eliminate various pollutants, including SMX.^21^

Graphitic carbon nitride (g-C_3_N_4_) with a bandgap of 2.7 eV is a promising photocatalytic material that has attracted significant attention due to its unique properties such as non-toxicity, chemical and thermal stability, low-cost precursors in the synthesis method and electronic properties suitable for photocatalytic applications.^22,23^ However, despite its potential, the photocatalytic performance of g-C_3_N_4_ is limited by several factors. One of the main challenges is the low quantum efficiency, which results in a low conversion rate of light energy into chemical reactions. Additionally, the fast recombination of electrons and hole pairs also hinders the photocatalytic performance of g-C_3_N_4_.^24–26^ Furthermore, the low surface area of g-C_3_N_4_ limits its interaction with target molecules, reducing the overall efficiency of the process.

Several methods have been developed to overcome these drawbacks and enhance the photocatalytic performance of g-C_3_N_4_. One approach involves the formation of porous structures, which increases the surface area and provides more active sites for chemical reactions.^27^ Another method is the modification of g-C_3_N_4_ with semiconductors to form heterojunctions, which can improve the separation of electrons and holes and enhance the photocatalytic properties.^28^ Doping elements into g-C_3_N_4_ is another strategy that has been explored to improve its performance. This can alter the electronic structure of the material and enhance its optical properties.^29^ Moreover, the formation of metal–organic framework structures and covalently organic frameworks has been proposed as an effective way to optimize the properties of g-C_3_N_4_ and enhance its photocatalytic activity.^30,31^

Overall, the development of these methods has provided new insights and opportunities for improving the photocatalytic performance of g-C_3_N, and further research in this field holds great promise for developing more efficient and sustainable energy conversion technologies.

Lanthanum ferrite (LaFeO_3_) photocatalyst, a perovskite-type oxide compound, is composed of iron, lanthanum and oxygen atoms arranged in a crystalline lattice with unique photophysical properties that have found applications in various areas, including solid oxide fuel cells, sensors, photocatalysts, and electrode materials. LaFeO_3_ possesses a small band gap ranging from 1.8 to 2.1 eV as well as good thermal and chemical stability, endowing it with great potential in the field of photocatalysis.^32,33^ Various researchers have shown that LaFeO_3_, with an orthorhombic perovskite structure, can decompose water.^34–36^

Upon the coupling/modification of LaFeO_3_ with g-C_3_N_4_ to form a heterojunction, which utilizes the adsorbed light over a wide spectral range and improves the photocatalytic performance, it also facilitates charge carrier separation and transfer, leading to enhanced photocatalytic efficiency. It has been shown that the coupling of LaFeO_3_ with g-C_3_N_4_ remarkably improves its catalytic activity for various applications.^34,37^

Numerous researchers have documented various studies utilising the use of sonophotocatalysis for the degradation of contaminants in water, such as N, Fe co-doped TiO_2_@SWCNT for the breakdown of sulfathiazole,^38^ MgO/CNT for the breakdown of sulfadiazine,^39^ and CuO–TiO_2_/rGO for the breakdown of methyl orange.^40^ Sonophotocatalysis relies on the physical cavitation phenomenon, enhanced by solid nanoparticles, to improve the contaminant decomposition efficiency using lower ultrasound intensities.^39^ The free radicals generated during the process effectively attack organic contaminants, particularly at the gas-bubble interface. Ultrasound-generated impulses prevent the aggregation of sonocatalyst nanoparticles, thus reducing the treatment time and energy consumption.

For this purpose, this study aims to synthesize LaFeO_3_@Pg-C_3_N_4_ heterojunction as an efficient sonophotocatalyst for the degradation of SMX in water. Therefore, LaFeO_3_ and LaFeO_3_@Pg-C_3_N_4_ were synthesized *via* a simple calcination/exfoliation method and characterized using UV-VIS, EDX, XRD, FTIR, SEM, and XPS techniques. The capacities of LaFeO_3_ and LaFeO_3_@Pg-C_3_N_4_ as efficient sonophotocatalysts for the degradation of SMX were compared under visible light irradiation. To circumvent the limitation of using g-C_3_N_4_ as a sonophotocatalyst, this study proposed that ultrasonic waves can generate surplus free hydroxyl radicals to facilitate effective charge transfer from LaFeO_3_ metal to g-C_3_N_4_ conduction band, leading to exceptional sonophotoactivity. Therefore, LaFeO_3_ was anchored on g-C_3_N_4_ to produce LaFeO_3_@Pg-C_3_N_4_ as an improved sonophotocatalyst for the degradation of SMX in an aqueous solution.

## 2 Materials and methods

### 2.1. Chemicals

Lanthanum oxide (La_2_O_3_) (≥99.9% Sigma-Aldrich), ferric nitrate nonahydrate (Fe(NO_3_)_3_·9H_2_O) (≥99.9% Sigma-Aldrich), hydrochloric acid (HCl), absolute ethanol (C_2_H_5_OH), sodium hydroxide (NaOH), melamine (C_3_H_6_N_6_), ammonium oxalate (C_2_H_8_N_2_O_4_), sulfamethoxazole, citric acid, nitric acid (≥65% Sigma-Aldrich), (C_28_H_31_ClN_2_O_3_), 1,4-benzoquinone (C_6_H_4_O_2_), isopropyl alcohol (C_4_H_10_O), humic acid, hydrogen peroxide, sodium chloride.

### 2.2. Synthesis of the materials

#### 2.2.1. Synthesis of the bulk graphitic carbon nitride

Melamine was used as the precursor; for this synthesis, 2 g of melamine was weighed and placed directly into a clean ceramic crucible, which was covered with a thin foil and transferred into a furnace. The melamine was heated for 4 h at 550 °C and the heating rate was kept at 3 °C min^−1^. The yellow-coloured material obtained was cooled and then subjected to grinding in an agate mortar to get a fine powdered form and it was labelled as B/g-C_3_N_4_.

#### 2.2.2. Synthesis of the porous graphitic carbon nitride

The finely ground bulk graphitic carbon nitride was transferred into a ceramic crucible and covered with tin foil. It was further subjected to heating for 4 h at 550 °C and the heating rate was kept at 3 °C min^−1^ in the furnace. The resulting-coloured yellow powder was cooled and stored in a container and labelled P/g-C_3_N_4_ nanosheet.

#### 2.2.3. Synthesis of lanthanum ferrite perovskite (La_0.8_F)

La_0.8_FO was synthesized according to the citric combustion route. 4.0264 g La_2_O_3_ (≥99.9% Sigma-Aldrich) was dissolved in a mixture of 5 mL deionized water and 10 mL HNO_3_ (≥65% Sigma-Aldrich) at a temperature of 100 °C till it dissolved. Then, 9.9854 g Fe(NO_3_)_3_·9H_2_O (≥99% Sigma-Aldrich) was added into the solution and stirred for about 5 min. Finally, 13.5042 g of citric acid monohydrate (≥99.0% Sigma-Aldrich) was added to the solution and stirred for 2 h. The solution was left to evaporate overnight at a temperature 120 °C to promote the gel formation, which was subsequently decomposed in the oven at 200 °C for 1 h. The obtained solid was ground and ultimately calcined at 700 °C at a flow rate of 6 °C min^−1^ for 6 h under static air. The resulting brick red powder obtained was stored in a container and labelled as (La_0.8_FO).

#### 2.2.4. Synthesis of lanthanum ferrite perovskite@P/g-C_3_N_4_ nanosheet (La_0.8_FO@PgNS)

La_0.8_FO@PgNS was synthesized through a wet impregnation-assisted ultrasonication method. A known amount of the synthesized P/g-C_3_N_4_ was measured and dispersed into a solution containing 30 mL methanol, stirred magnetically for 20 h, and further sonicated for 2 h. A known weight of the synthesized La_0.8_FO was added into the mixture of methanol and P/g-C_3_N_4_, stirred for 5 h and sonicated for 1 h. The obtained reddish-brown powder was called La_0.8_FO@PgNS.

### 2.3. Characterization of the as-synthesized sonophotocatalyst

The photocatalyst was analyzed on an FTIR spectrometer (Shimadzu 8400S) at 500–4000 cm^−1^ to determine the functional moieties present in the synthesized materials. The sample was prepared for FTIR using KBr. Powder X-ray diffraction (XRD) patterns were acquired with a Bruker D8 Advance diffractometer (Billerica, MA, USA) in Bragg–Brentano geometry, employing a Cu-Kα radiation source (*λ* = 0.154 nm), powered at 40 kV and 40 mA. Scanning electron microscopy (SEM) images were acquired with a Zeiss SUPRA 40 V P microscope (Zeiss, Oberkochen, Germany), setting the electron acceleration voltage at 5 or 10 kV. Energy-dispersive X-ray analysis (EDX) was coupled with SEM for elemental quantification at 20 kV electron acceleration voltage. X-ray photoelectron spectroscopy (XPS) was performed with a Thermo Scientific ESCALAB QXi spectrometer (Waltham, MA, USA), employing a monochromatized Al-Kα source (*hν* = 1486.68 eV) and a charge compensation gun (cluster type). Elemental quantification was carried out by the integration of La 3d_5/2_, Fe 2p, O 1s, C 1s, and N 1s photopeaks after Shirley-type background subtraction. The SMX concentration was measured using a UV-vis spectrophotometer (1800, Shimadzu, Japan). UV-DRS was performed using a LAMBDA 1050 UV/vis spectrophotometer (PerkinElmer, Waltham, USA) equipped with a solid sample reflectance kit and BaSO_4_ as the reference standard, and the optical bandgap energy of the nanocomposite photocatalyst was estimated using the Tauc plot equation through the Kubelka–Munk function.

### 2.4. Sonophotocatalytic degradation of SMX

The optimization of the sonophotocatalytic activities of La_0.8_FO@PgNS material was carried out through the oxidation of SMX antibiotics in water. To maximise the adherence of antibiotic particles on the catalyst surface, a solution of 50 mL of a known concentration of 5.00 mg L^−1^ of SMX and 0.10 g sonophotocatalyst was stirred in the dark for 30 min before the sonophotocatalytic degradation experiment. The catalytic degradation reaction was conducted using an ultrasonication instrument with a 300 W Xenon lamp (Xe Ozone free-6258) emitting in the UV to visible spectrum (>420 nm). An aliquot of the sample was isolated from the reaction system every 20 min during the process. After centrifugation and filtration, the absorbance of the solution was measured at the maximum absorption wavelength of 257 nm using a UV-visible spectrophotometer.

The sonophotocatalytic degradation process was investigated to determine the impact of various operational parameters, including concentration (ranging from 1.00 to 5.00 mg L^−1^), weight (ranging from 0.01 to 0.20 g), pH (ranging from 2 to 10), the effect of anions (1 mM bicarbonate, sulphate and phosphate, the effect of natural organic matters (humic and fulvic acid)), the effect of oxidant (0.5% H_2_O_2_, 1% H_2_O_2_), the effect of radical trappers (ammonium oxalate, isopropyl alcohol, benzoquinone). The percentage degradation was calculated using eqn (1) below.1
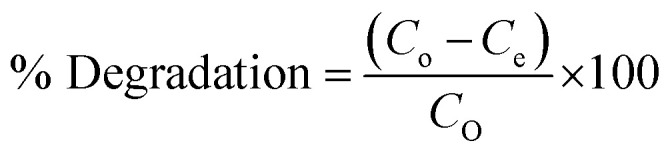
*C*_o_ is the initial concentration of the organic contaminants in mg L^−1^, and *C*_e_ is the equilibrium concentration of the organic contaminant in mg L^−1^.

The degree of mineralization in water with the prepared sonophotocatalysts was determined *via* the measurement of the oxygen equivalent of the organic matter present in each sample, for example, total organic carbon (TOC), using a Shimadzu TOC Analyzer.

The experiments were conducted thrice and the values were recorded as the mean of triplicate measurements.

The reactive species generated by the sonophotocatalyst La_0.8_FO@PgNS was confirmed by performing the scavenger test using 1 mM ammonium oxalate (AO) as the positive hole scavenger, benzoquinone (BQ) as the superoxide scavenger, and isopropyl alcohol (IPA) as the hydroxyl radical scavenger.

At the end of each sonophotocatalytic degradation process, La_0.8_FO@PgNS was recovered from the solution by centrifugation, washed with water and dried at 80 °C for 2 h and then used for further tests.

## 3 Results and discussion

### 3.1. Characterization

#### 3.1.1. Fourier transform infrared spectroscopy

**Fig. 1:**
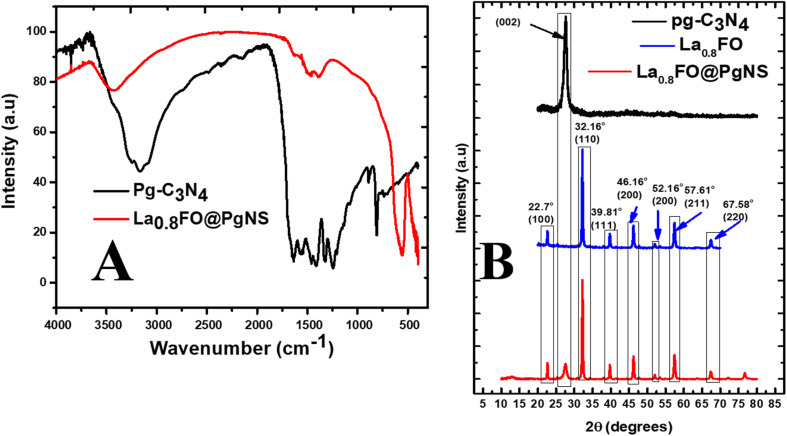
(A) Fourier transform infrared spectra of Pg-C_3_N_4_ and La_0.8_FO/PgNS. (B) X-ray diffraction patterns of Pg-C_3_N_4_, La_0.8_FO and La_0.8_FO@PgNS.

Fig. 1A exhibits the FT-IR spectra of Pg-C_3_N_4_ and La_0.8_FO@PgNS in the range of 400–4000 cm^−1^. The functional moieties present in the prepared material were confirmed through the FT-IR spectrum. The stretching mode of the OH group of water molecules adsorbed on the surface of Pg-C_3_N_4_ or the N–H group in an uncondensed state was suggested by the FTIR peak centered at 3419, 3167 cm^−1^.^22,25^ The bending vibration of the hydroxyl of water attached to the surface of Pg-C_3_N_4_ was provided by the peak available at 1637 cm^−1^. C–N and C=N groups of polymerized Pg-C_3_N_4_ heterocycles were suggested by some other peaks found in the range of 1571–1255 cm^−1^,^23^ while for La_0.8_FO@PgNS, the peak shifted to 1630 and 1459 cm^−1^ for the C–N and C=N groups. The significant reduction in the intensity of the C–N and C=N peaks of Pg-C_3_N_4_ in the modified sample La_0.8_FO@PgNS can be attributed to a lower amount of Pg-C_3_N_4_ in the sample. The *S*-triazine breathing mode was suggested by a very strong peak at 805 cm^−1^.^28^ The absorption band in the range of 540–570 cm^−1^ for La_0.8_FO@PgNS suggested the formation of M–O bonds, where M = La and Fe, and the La–O and Fe–O stretching vibrations corresponded to octahedral LaO_6_ and FeO_6_ groups present in perovskites.^41–44^

#### 3.1.2. X-ray diffraction

The crystal–phase structure of Pg-C_3_N_4_ and La_0.8_FO@PgNS was analyzed using XRD, as shown in Fig. 1B. The crystal plane (100) was attributed to the layered stacking structure and the crystal plane (002) formed by the aromatic stacking units (PDF 00-087-1526). The diffraction peaks of La_0.8_FO were available at 2*θ* values of 22.7°, 32.16°, 39.81°, 46.16°, 52.16°, 57.61° and 67.58°,respectively, matching the crystal planes (100), (110), (111), (200), (210), (211) and (220), which corresponded to the orthorhombically distorted perovskite structure (PDF 01-089-1268). The diffraction peaks of La_0.8_FO@PgNS exhibited the characteristic peaks of Pg-C_3_N_4_ and La_0.8_FO, similar to those of La_0.8_FO and Pg-C_3_N_4_, confirming no impurity XRD peak and signifying that the product prepared has superior purity. Also, the reduction of the characteristic Pg-C_3_N_4_ peak at 27.1° was observed to be devoid of peak broadening, which confirms the retention of its crystallinity; similar trends were observed in the report of Ismael and his colleagues, which can be attributed to the lower amount of Pg-C_3_N_4_ in the sample.^35^ The Scherrer equation was used to calculate the crystalline size of La_0.8_FO and La_0.8_FO@PgNS.2
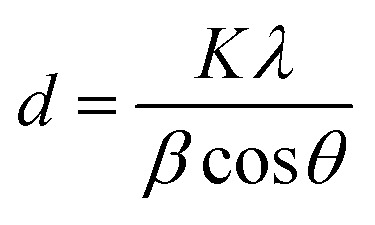
where *λ* is the wavelength, *β* is the full width at half maximum, *θ* is the Bragg's angle for the given diffraction and *D* is the crystallite size.

The crystallite size was estimated to be 28.35 nm and 34.20 nm from the most intense peak of the (110) plane.

#### 3.1.3. Scanning electron spectroscopy and energy dispersive X-ray analysis

**Fig. 2:**
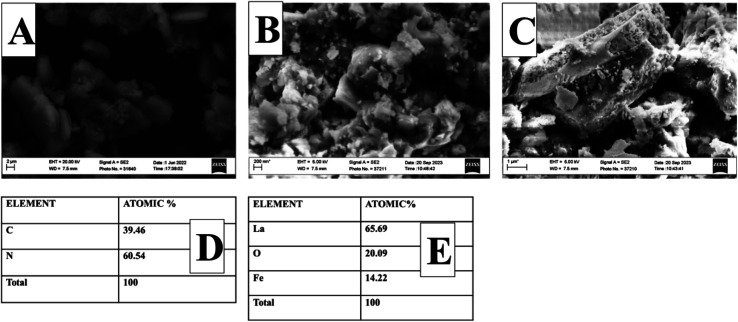
Scanning electron microscopy images of (A) Pg-C_3_N_4_, (B) La_0.8_FO, (C) La_0.8_FO/PgNS. Energy dispersive X-ray analysis of (D) Pg-C_3_N_4_ and (E) La_0.8_FO.

The surface morphology of the as-synthesized materials is shown in Fig. 2A, which shows the image of Pg-C_3_N_4_, having a typical layered platelet-like morphology with a large amount of agglomeration on the surface, while Fig. 2B shows that the morphology of La_0.8_FO has a mixed size grains with irregular shapes. Fig. 2C shows the morphology of the modified material La_0.8_FO@PgNS; it was observed that the irregularly-sized La_0.8_FO microparticles were attached to Pg-C_3_N_4_.

Elemental analysis was performed to study the elemental distribution in the material Pg-C_3_N_4_, as seen in Fig. 2D; the presence of C (39.46%) and N (60.54%) elements in Pg-C_3_N_4_ confirmed that no impurities were present in it, as expected. Also, the presence of O (65.69), La (20.09), and Fe (14.22), as shown in Fig. 2E, confirmed the successful synthesis of La_0.8_FO without impurities.

#### 3.1.4. X-ray photoelectron spectroscopic analysis

**Fig. 3:**
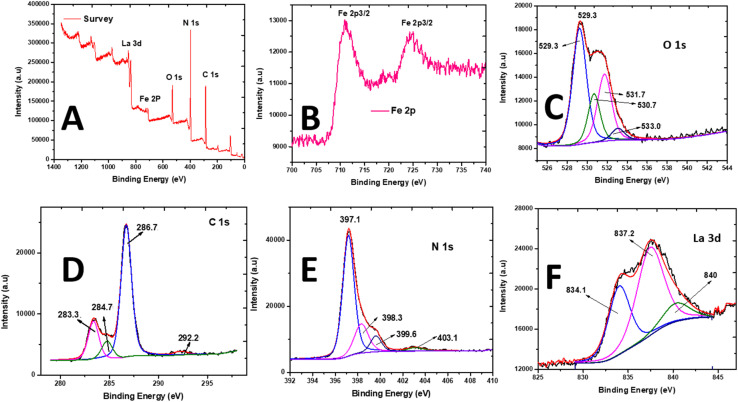
X-ray photoelectron spectroscopy spectra of La_0.8_FO/PgNS. (A) Scanning survey, (B) Fe 2p photopeak, (C) O 1s photopeak, (D) C 1s photopeak, (E) N 1s photopeak, and (F) La 3d_5/2_ photopeak.

The chemical composition and oxidation states of the elements in La_0.8_FO/PgNS were determined through X-ray photoelectron spectroscopy (XPS) analysis (Fig. 3). The XPS survey spectrum revealed the presence of La, Fe, O, N, and C elements, as shown in Fig. 3A.

The peaks of Fe 2p_3/2_ and Fe 2p_1/2_ were observed at 710.9 and 724.0 eV, respectively, indicating the presence of Fe^3+^ cations in the oxide (Fig. 3B). The O 1s peak was deconvoluted into four signals located at 529.2, 531.7, 530.7, and 533.1 eV, which were attributed to the perovskite M–O bond (La–O and Fe–O bonds) and chemisorbed O-containing species (surface-adsorbed hydroxyl and carbonate groups ˙OH and CO_3_, respectively) on the surface of La_0_._8_FO (Fig. 3C).^45^

The C 1s spectra were deconvoluted (Fig. 3D), revealing the presence of peaks at 283.4 and 286.7 eV, which were due to the sp^2^ hybridized C atoms (*i.e.*, N–C=N, C=C) and graphitic-carbon (*i.e.*, C–N) from the contaminated carbon on the surface of La_0_._8_FO/PgNS and sp^2^ C atoms bonded to the amino groups in the triazine cycles, respectively.^34^ The peak at 284.8 eV was accredited to the sp^3^ hybridization of C atom, while the peak at 292.3 eV was accredited to the π–π* satellite.^46^ The N 1s spectrum (Fig. 3E) showed peaks at 397.2, 398.3, and 403.1 eV, attributed to the sp^2^-hybridized N atoms (C–N=C) in the heptazine rings, tertiary N atoms (N–(C)_3_), and C–N–H, respectively.^34,47^ The N 1s and C 1s spectra confirmed the presence of g-C_3_N_4_.

The XPS spectrum of La 3d_5/2_ showed two main contributions at 834.0 and 837.2 eV (Fig. 3F). The binding energies confirmed that La was present in the +3 oxidation state and incorporated in an oxide. These peaks were produced by the transfer of an electron from the 2p to the empty 4f orbital in the O_2_ ligands.^48,49^ XPS analysis proved that La_0.8_FO and Pg-C_3_N_4_ had strong interactions with each other, confirming the formation of La_0.8_FO@PgNS.

The surface comparison between the XPS and EDX composition (Table 1) confirmed the presence of La, N, O, Fe and C. These results also confirmed the successful synthesis of La_0_._8_FO/PgNS as the sonophotocatalyst.

**Table 1:** XPS and EDX surface composition of La_0.8_FO@PgNS

Element	XPS atomic%	EDX atomic%
N 1s	41.8	39.28
C 1s	39.8	39.8
O 1s	13.5	17.78
La 3d	2.4	1.50
Fe 2p	2.5	1.64
Total	100	100

#### 3.1.5. UV-DRS and Tauc plot

**Fig. 4:**
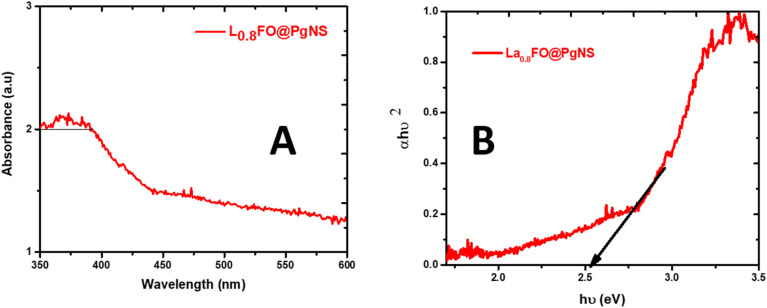
(A) UV-visible diffuse reflectance absorption spectrum and (B) estimated energy band gap spectra of the La_0.8_FO@PgNS sonophotocatalyst.

Fig. 4A illustrates the UV-vis DRS of La_0.8_FO/PgNS, which displays an absorption edge at 682 nm^50^ (as seen in Fig. 4B). This result is consistent with previous findings in several studies. The optical bandgap (*E*_g_) was determined using the Tauc/David-Mott model, which involved plotting (*αhν*)^1/*n*^*vs. hν*, where *h* is Planck's constant, *α* is the absorption coefficient, *ν* is the photon frequency, and *n* is 1/2 for direct allowed transitions or 2 for indirect allowed transition;^45,51,52^ in our case, the *n* is 1/2. To estimate the *E*_g_ value, the linear region of the plot was extrapolated onto the energy axis, and it was found to be 2.52 eV. This means that the material absorbs light in the visible spectrum. In perovskite-type oxide materials, the transition of electrons between the valence band (O 2p) and the conduction band (Fe 3d) is primarily responsible for the strong absorption edges.^53,54^

## 4 Degradation test

**Fig. 5:**
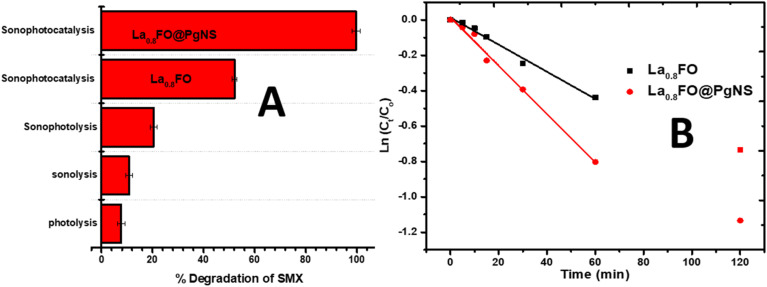
(A) Degradation performance of SMX using photolysis, sonolysis, sonophotolysis and sonophotocatalysis using La_0.8_FO and La_0_._8_FO@PgNS. (B) Pseudo first-order kinetic model of the sonophotocatalysis data using La_0_._8_FO and La_0_._8_FO@PgNS.

The performance of the different components of the as-synthesized material and the role of sonication and photolysis (visible light) in the removal of SMX were investigated by performing some confirmatory preliminary tests. It was observed that the very low removal efficiency of SMX was observed using photolysis (visible light) (7.698%), sonolysis (10.753%) and sonophotolysis (20.436%) irradiations in the absence of the catalyst within 120 min reaction time, as seen in Fig. 5A.

For sonophotocatalysis, a suspension of the catalyst La_0_._8_FO/PgNS and SMX was stirred for 30 min under the dark condition to reach the adsorption–desorption equilibrium. 9.821% and 16.716% SMX degradation was observed after 30 min of stirring in the presence of La_0_._8_FO and La_0_._8_FO/PgNS, respectively. On the other hand, when both ultrasound and visible light irradiation were combined in the presence of the different catalysts La_0_._8_FO and La_0_._8_FO/PgNS, the efficiency was observed to be 52.063 and 99.603% in 120 min, respectively, as compared to the performance of sole sonolysis (10.753%) in 120 min (Fig. 5A). The result proved that the modification of La_0_._8_FO with Pg-C_3_N_4_ improved the degradation efficiency of the catalyst and this can be attributed to the lowering of the electron–hole pair recombination of the sono/photogenerated charge species, leading to the increment of their lifetime. Pg-C_3_N_4_ was evenly dispersed into La_0_._8_FO during the incorporation process, which resulted in the availability of more active sites, which in turn led to the production of more reactive species that consequently increased the degradation efficiency of La_0_._8_FO/PgNS for SMX. Also, the ultrasonic wave produced more cavitation energy in the presence of the solid catalyst due to the lower solid–liquid tensile strength, which transforms into microbubbles, thus efficiently producing higher localized pressure and temperature that break down the water molecule (water dissociation) and produce hydroxyl radicals; in the presence of the hydroxyl radical and superoxide, the degradation process is enhanced.^21,55,56^ Also, the sonoluminesence can excite the semiconductor.^57^

The SMX sonophotocatalytic degradation was evaluated kinetically (eqn (3)); the results shown in Fig. 5A were fitted to the pseudo-first-order kinetic model and are shown in Fig. 5B.

3 ln[*C*_*t*_/C_o_] = −*K*_1_*t*

where *k*_1_ is the rate constant (min^−1^), *C*_o_ is the equilibrium concentration of (mg L^−1^), and *C*_*t*_ is the concentration of SMX at time *t* (min).

The rate constants (*k*_app_) for the degradation reaction were estimated, as presented in Table 2.

**Table 2:** Kinetic parameters for the photocatalytic degradation of SMX using La_0.8_FO and La_0.8_FO/PgNS

Sono-photocatalyst	*k*_app_ (min^−1^)	*t*_1/2_ (min)	*r* ^2^
La_0.8_FO	0.01343	51.600	0.9874
La_0.8_FO@PgNS	0.01494	46.385	0.9900

The maximum *K*_app_ observed was achieved using La_0_._8_FO@PgNS sonophotocatalyst for the degradation of SMX. This indicates the significant role of Pg-C_3_N_4_ in reducing the electron–hole recombination rate.

The study examined the effectiveness of combining different techniques such as photolysis, sonolysis, sonophotolysis and sonophotocatalysis for the degradation of SMX, a widely used antibiotic. The researchers estimated the synergistic effect of combining these techniques using a formula (eqn (4)) and found that the combination resulted in a 2.5-fold synergy, indicating a highly effective approach.4
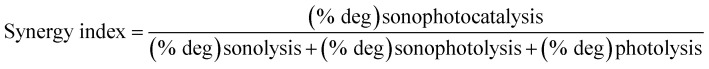


The synergistic index value is greater than 1, indicating that the efficiency of the sonophotocatalytic degradation is higher than the cumulative value of the individual processes (photolysis, sonocatalysis or sonophotocatalysis). The high synergy observed can be attributed to the various effects occurring during the process; the ultrasound wave played a significant role in maintaining a high dispersion of the catalysts La_0.8_FO and La_0.8_FO/PgNS, leading to increased collision and adsorption–desorption process. The visible light-mediated photodecomposition of H_2_O_2_, produced by the sonolysis of water, was also found to be effective in degrading SMX.^58,59^ Additionally, the hydroxyl radical generated by sonolysis and photocatalysis systems contributed to the combined degradation effect.^60^ The study concludes that the synergistic effect of combining these techniques holds great promise for the efficient degradation of antibiotics in wastewater treatment.

**Fig. 6:**
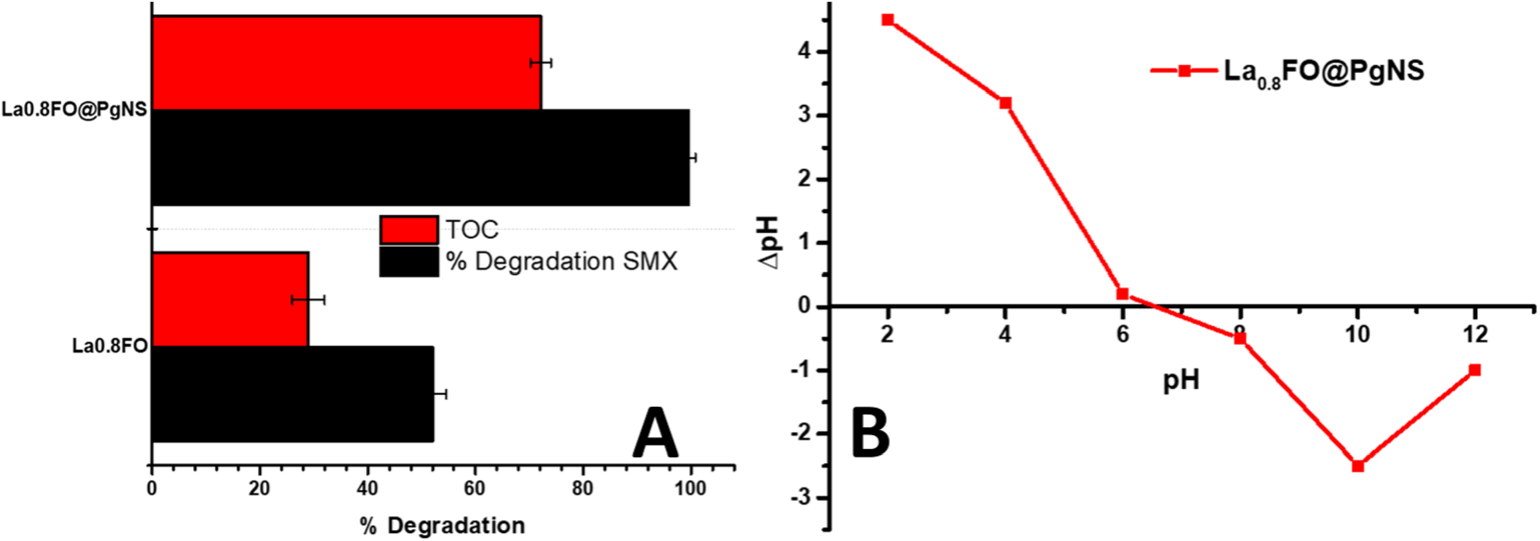
(A) The % degradation of SMX juxtaposed against the % TOC removal (mineralization), and (B) pHPZC of La_0.8_FO@PgNS.

Furthermore, the sonophotocatalytic degradation ability of La_0.8_FO and La_0.8_FO@PgNS was evaluated by measuring the TOC values of SMX working solution before and after the experiment, which gives us an insight into the degree of mineralization of the contaminants into CO_2_ and H_2_O. TOC removal (%) was observed in the range of 28.99 to 72.10%, as shown in Fig. 6A.

### 4.1. Effect of operational variables

This study investigated the effects of various operational parameters on the sonophotocatalytic degradation of SMX using La_0.8_FO@PgNS as a catalyst. The parameters considered include the initial concentration of SMX, sonophotocatalyst dose, pH, anion type, natural organic matter (NOM), ionic strength, ultrasonic power, and the effect of the oxidant (H_2_O_2_), using La_0_._8_FO@PgNS the most effective sonophotocatalyst for SMX degradation.

**Fig. 7:**
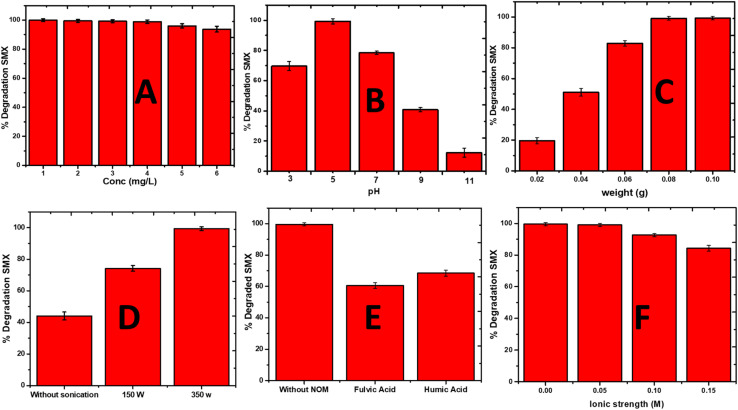
The effect of (A) initial concentration, (B) initial solution pH, (C) sonophotocatalyst dose, (D) ultrasonic power, (E) natural organic matter, and (F) ionic strength on the degradation of SMX using La_0.8_FO@PgNS.

The degradation efficiency of La_0_._8_FO@PgNS decreased from 100% to 93.78% in 120 min as the initial concentration of SMX was increased from 1 ppm to 6 ppm, as shown in Fig. 7A. This decrease in efficiency could be attributed to the increased availability of SMX species in the solution, requiring more capacity of La_0_._8_FO@PgNS for the degradation process. Additionally, higher concentrations of SMX required a higher amount of reactive oxygen species (ROS) to degrade the pollutant. Consequently, the same amounts of ROS generated under the same operational conditions led to lower degradation efficiency with the increased SMX concentration.

The pH is a crucial factor in the degradation of SMX in water as it can affect the particle size and the degree of aggregation of the sonophotocatalyst molecule, which alters the rate of degradation of organic molecules in water.^61,62^ The solution pH was varied employing the use of HCl and NaOH; the change in pH caused a change in the charge distribution on the surface of the sonophotocatalyst.^63^ The charge distribution on the surface of the sonophotocatalyst La_0.8_FO/PgNS was determined by the salt-addition method analysis at different pH values.^63^

The pH_pzc_ value of La_0.8_FO/PgNS was observed to be 6.52, as shown in Fig. 6B. The pH_pzc_ is the point at which the surface of the catalyst carries zero charge. Below the pH_pzc_ value, the surface becomes positively charged while above the pH_pzc_, the surface becomes negatively charged.^63^ The pH level of the solution affects the charge of the material's surface and the ionization state of sulfamethoxazole (SMX), which in turn impacts the degradation efficiency. When the pH is below 6.52 in acidic conditions, SMX (with *p*K_a1_ of about 1.6 and *p*K_a2_ of about 5.6) mainly exists in its neutral or negatively charged form (anion) within this pH range. We observed that the degradation efficiency increased from pH 3 (69.59%) to pH 5 (99.32%) due to the enhanced electrostatic attraction between the positively charged surface and the negatively charged SMX molecules. This strong interaction led to high degradation, facilitated by strong adsorption, thereby promoting the greater interaction and breakdown of the SMX molecules. There was a slight decrease in the efficiency at pH 7 (78.48%) as SMX exists in its neutral form and is primarily driven by non-electrostatic interactions such as van der Waals forces or hydrophobic interactions. For pH levels above 6.52, the material surface becomes negatively charged, and SMX exists in its anionic form. The efficiency decreases due to weaker or repulsive electrostatic interactions, resulting in lower adsorption and therefore lower degradation rates, as observed in our experiment (Fig. 7B).^64,65^

As the weight increased from 0.01 to 0.1 g, the degradation of SMX by La_0.8_FO/PgNS increased; the increment in the degradation efficiency may be attributed to the availability of more active sites as the surface area available for sonophotocatalytic degradation increased with the increased weight of La_0_._8_FO@PgNS (Fig. 7C). A similar observation was recently reported by Adewuyi and his colleague.^28^

The effect of ultrasonic power was optimized to get a clearer picture of how it influences the reaction. Increasing the ultrasonic power from 150 to 350 W led to a higher SMX degradation efficiency; this is due to the increase in the production of the cavitation microbubble, which in the end collapse and leads to the generation of more reactive oxygen species because of sonoluminesence and sonolysis enhancing the excitation of the catalyst (Fig. 7D).^20,55^ The optimum degradation efficiency was observed when the ultrasonic intensity was increased to 350 W; due to the increased turbulence in the solution, mass transfer was aided.^66^

The presence of NOM (1 mg L^−1^ fulvic and humic acid), a model NOM usually found in some water^67^ and ionic strength (NaCl with the strength ranging from 0.05 to 0.15 M), it was observed that the presence of humic substances reduced the degradation efficiency from 99.52% without NOM to 60.47 and 68.41% in 120 min, in the presence of humic and fulvic acid, respectively (Fig. 7E). This decrease in the efficiency could be due to the NOM competing with the SMX for the reactive species and the active sites available for the degradation process.^68,69^

The study found that higher concentrations of ionic strength (0.15 M) resulted in lower degradation efficiency (Fig. 7F). This decrease can be attributed to the blocking of the active site by the chloride ions, which have been proven to be the positive hole scavengers, preventing the formation of hydroxyl radicals, which is needed for the degradation of SMX, as seen in eqn. (5) and (6) below.^70^

5 Cl^−^ + OH˙ → Cl˙ + OH^−^

6 Cl˙ + Cl^−^ → Cl_2_˙^−^

**Fig. 8:**
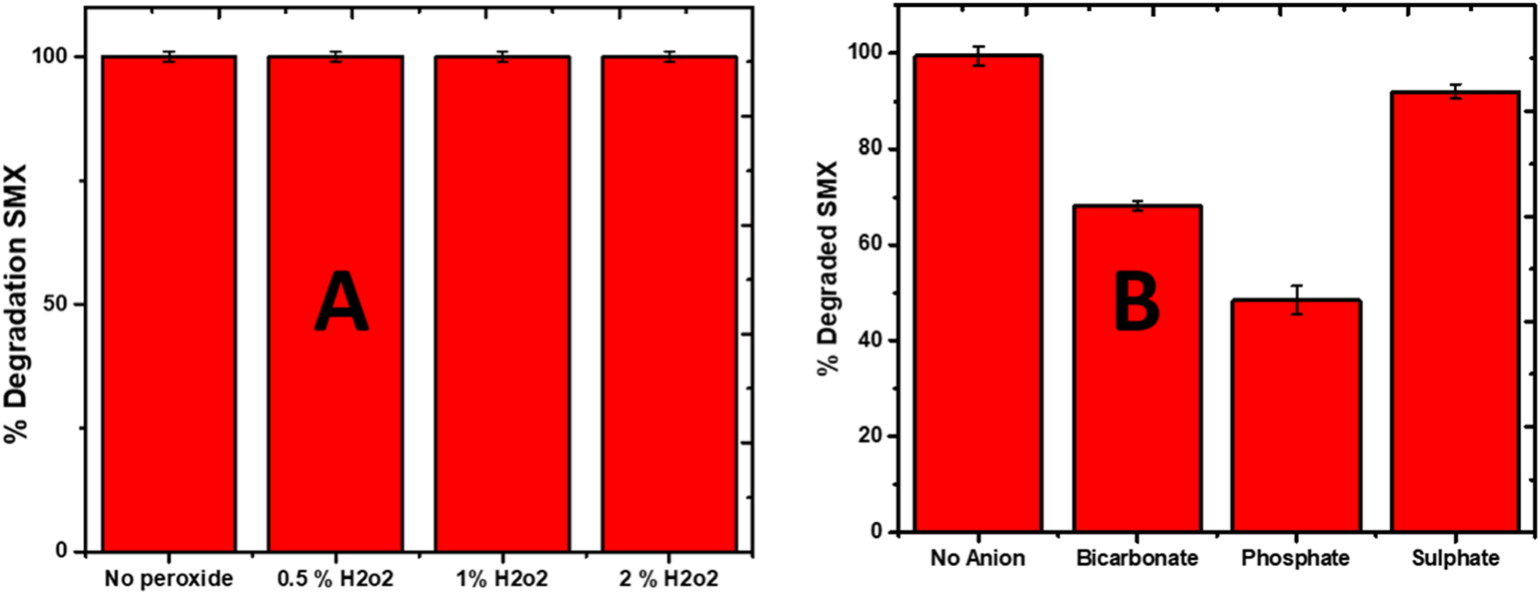
The effect of (A) oxidant (H_2_O_2_) and (B) anion on the degradation of SMX using La_0.8_FO@PgNS.

The effect of the oxidant (H_2_O_2_) was also investigated, and it was found that the addition of H_2_O_2_ significantly enhanced the degradation efficiency of SMX. The addition of different percentages (0.5, 1 and 2%) if H_2_O_2_ raised the degradation efficiency of La_0_._8_FO/pg for SMX to 100% in 25 min (Fig. 8A). It is believed that H_2_O_2_ in photocatalytic systems generated additional HO˙ *via* the following mode, as seen in eqn (7)–(9).

7 H_2_O_2_ + e^−^ → HO˙ + OH^−^

8 H_2_O_2_ + *hv*^+^ → 2HO˙

9 H_2_O_2_ + O_2_˙^−^ → HO˙ + OH^−^ + O_2_

The effect of different anions (bicarbonate, sulphate, and phosphate) was investigated, and it was observed that bicarbonate and phosphate decreased the degradation efficiency of La_0_._8_FO/PG for SMX (Fig. 8B). Both have been reported to be hydroxyl radical scavengers, as seen in eqn (10)–(12).

10 CO^2−^_3_ + OH˙ → CO_3_˙^−^ + OH˙^−^ + O_2_

11 CO_3_˙^−^ + CO_3_˙^−^ → CO_2_ + CO_4_^2−^

12 SO_4_^2−^ + OH˙ → SO_4_˙^−^ + OH˙

In conclusion, the study provides insights into the optimized experimental conditions for the sonophotocatalytic degradation of SMX using La_0_._8_FO/PgNS. This result confirmed that it could be useful for the design and optimization of sonophotocatalytic systems for the treatment of SMX-contaminated water.

### 4.2. Scavenging test

**Fig. 9:**
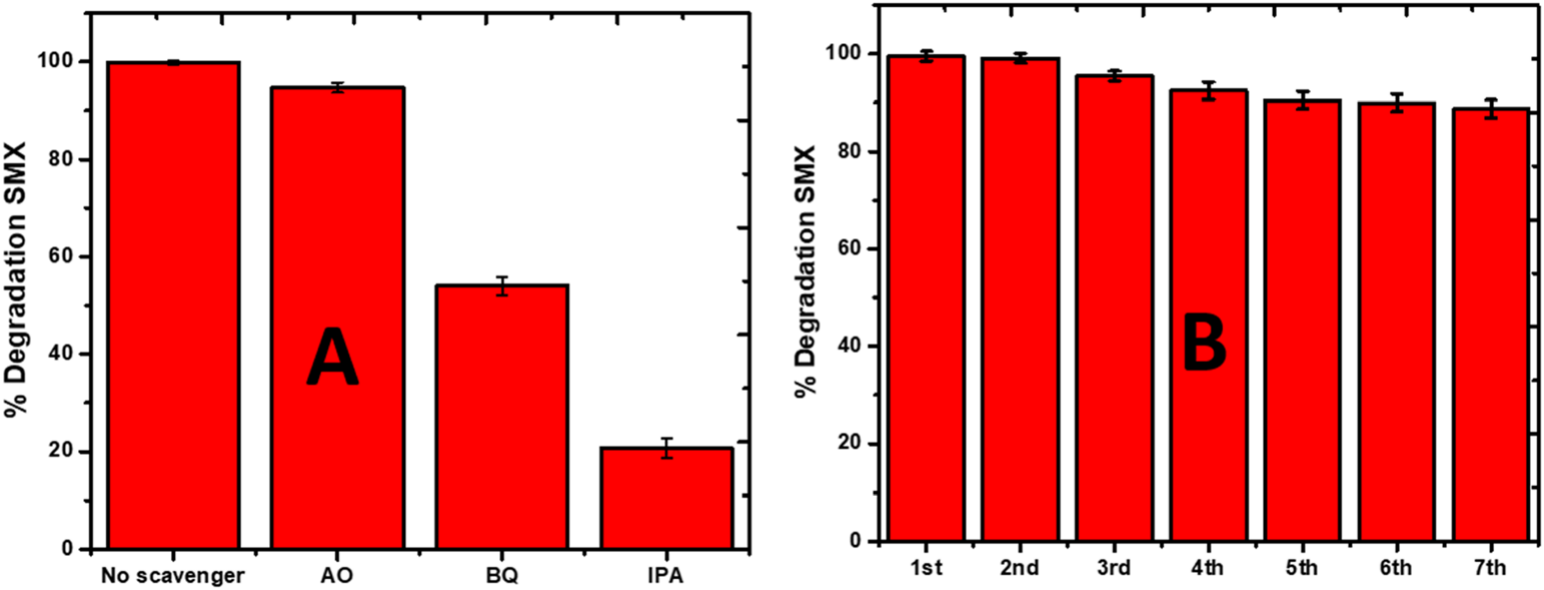
(A). Effect of scavengers (isopropanol-IPA, benzoquinone-BQ, and ammonium oxalate-AO) on the performance of the sonophotocatalyst La_0_._8_FO@PgNS for the degradation of SMX. (B) Reuse efficiency of the La_0_._8_FO@PgNS sonophotocatalyst for the degradation of SMX.

To estimate the role that different reactive oxygen species played in the degradation process, the scavenger test was performed using 1 mM ammonium oxalate (AO) as the positive hole scavenger, benzoquinone (BQ) as the superoxide scavenger, and isopropyl alcohol (IPA) as the hydroxyl radical scavenger. These scavengers were used to predict the plausible degradation mechanism of SMX by sonophotocatalysis over La_0_._8_FO@PgNS, as shown in Fig. 9A. The sonophotocatalytic activity was not inhibited for AO with an efficiency of 94.68%, while the activity was inhibited for BQ and IPA, suggesting that the hydroxyl and superoxide radicals are the leading active species involved in the sonophotocatalytic degradation of SMX in the order ˙OH > ˙O_2_^−^ > h^+^.

### 4.3. La_0_._8_FO@PgNS reusability and application in real water samples

The practical applicability of the La_0_._8_FO/PgNS sonophotocatalyst was investigated to determine its reusability potential. To achieve this goal, the composite was used in seven subsequent cycles, and the degradation efficiency of SMX was monitored after each experimental cycle. The sonophotocatalyst was washed with Millipore water to remove any contaminant residue and dried after each cycle. In the first four cycles, no significant loss was observed, indicating that the catalyst is highly durable (Fig. 9B). However, it is worth noting that about a 10% decrease in the degradation efficiency of SMX was observed at the end of the seventh cycle experiment, indicating that some reduction in the catalyst's efficiency occurred after multiple uses. Despite this, no mass loss was observed after subsequent usage as the composite could be easily removed from the treated water and fully recycled. These results confirm the sustainability of the La_0_._8_FO@PgNS sonophotocatalyst and its potential for practical applications.

The results obtained from the current study were compared with previously reported photocatalysts for the degradation of SMX, as shown in Table 3, and it proved to be a very efficient material for SMX degradation.

**Table 3:** Comparison with other reported catalysts for the degradation of SMX in water^a^

Catalyst	Contaminant	Degradation efficiency	References
NdFe_2_O_4_@g-C_3_N_4_	CIP	100.00	28
	AMP	96.80	
SnFe_2_O_4_@monoZIF-8	SMX	100.00	64
	CIP	100.00	
	AMP	100.00	
	ERY	91.00	
g-C_3_N_4_@ZnO	SMX	94.20	71
Ag_2_S/Bi_2_S_3_/g-C_3_N_4_	SMX	97.40	72
ZrFe_2_O_4_@ZIF-8	SMX	100.00	65
	DOP	100.00	
PAA/AC600	SMX	93.70	73
Biochar/TiO2	SMX	91.00	74
Ti/C–N–TiO_2_	SMX	99.99	75
La_0_._8_FO	SMX	51.60	This study
La_0_._8_FO/PgNS	SMX	99.60	This study

a Ciprofloxacin = CIP, ampicillin = AMP, PAA = peracetic acid, AC = activated carbon, Ag_2_S = silver sulfide, Bi_2_S_3_ = bismuth(iii)sulfide, and Ti/C–N–TiO_2_ = carbon–nitrogen co-doped catalysts.

La0.8FO@PgNS was applied to degrade SMX in real water samples. The study used water samples (Table 4) spiked with SMX. The results showed that La_0.8_FO@PgNS effectively degraded SMX in the water samples. The removal efficiency of SMX was higher in Millipore water (99.60%) compared to tap and wastewater (93.46% and 81.5%), respectively. This difference may be due to the presence of chlorine and anions in tap water and a complex matrix of organic compounds and dissolved inorganic substances in the wastewater sample. This suggests that La_0.8_FO@PgNS could potentially be used for the remediation of other pharmaceuticals in real water samples.

**Table 4:** Physical water quality parameters of the water samples

Parameter	Tap water	Waste water
pH	7.19	4.38
TDS (mg L^−1^)	240	654
Temperature (°C)	21.1	22.6
Resistivity (Ω)	324	720
Conductivity (mS)	614	989.9
Redox (mV)	44.7	92.6

### 4.4. Plausible mechanism of sonophotocatalytic degradation

**Fig. 10:**
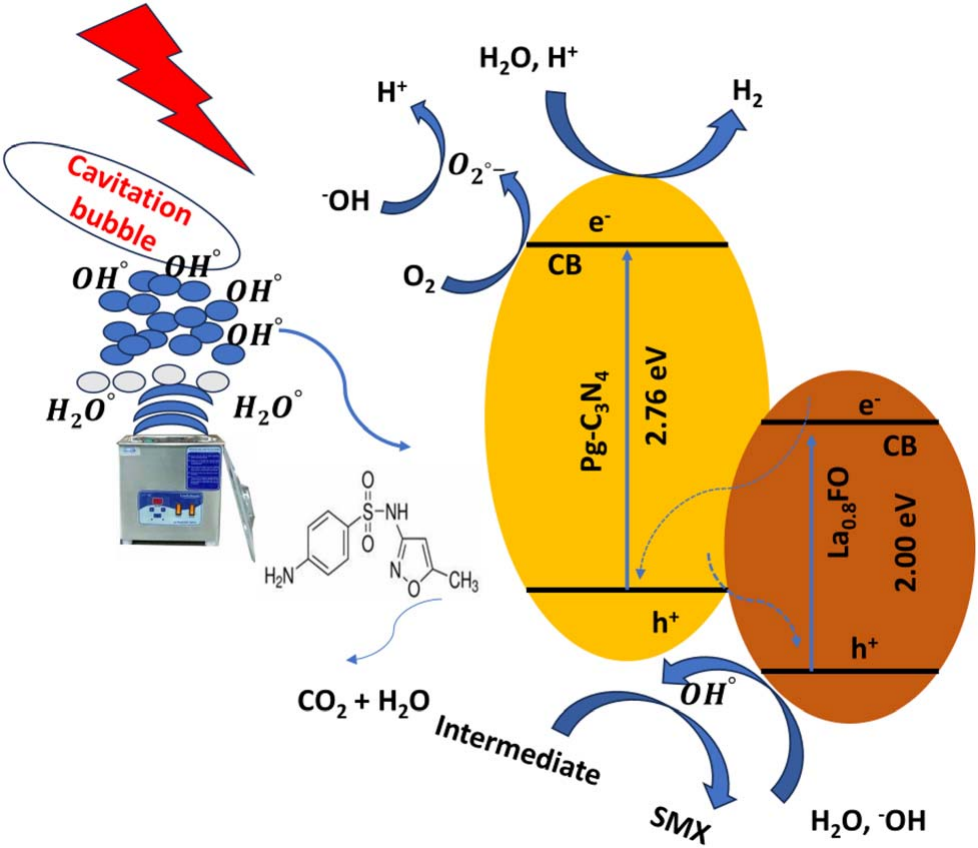
Possible mechanism for the sonophotocatalytic degradation of SMX over the Z-La_0_._8_FO@PgNS.

Based on the experimental results, a possible degradation mechanism was proposed. The La_0_._8_FO@PgNS heterojunction system was schematically represented, depicting the activities of reactive oxygen species generation, charge separation, transfer, and sonophotocatalysis. La_0.8_FO coupled with Pg-C_3_N_4_ forms a heterojunction (Fig. 10).

The sonolysis of water could produce cavitation bubbles with very high pressure and temperature, leading to the breakdown of water molecules to generate hydroxyl and hydrogen radicals. The generated cavitation bubbles from sono-irradiation could physically affect the mass transfer of SMX to the surface of La_0_._8_FO@PgNS from the solution. H_2_O_2_ is a strong oxidizing agent, and its presence during the sonophotocatalytic process could play a significant role in influencing the performance of the degradation of SMX by dissociating into hydroxyl radicals under ultrasonic irradiation, leading to the production of abundant free radicals for SMX degradation.

When exposed to light, electron–hole pairs were produced, leading to the stimulation of electrons in both components to their respective conduction bands. Meanwhile, holes continued to appear in their corresponding valence bands. The proximity of the conduction band of La_0.8_FO and the valence band of Pg-C_3_N_4_ led to the recombination between the electrons of La_0.8_FO and positive holes of Pg-C_3_N_4_.^76,77^

The photo-excited electrons of La_0.8_FO and Pg-C_3_N_4_ transitioned from the valence band to the conduction band, while the holes were left on the valence band. The electrons from the conduction band of La_0.8_FO transferred to the valence band of Pg-C_3_N_4_ through the solid–solid intimately contacted interfaces. The electrons and holes recombined, thus enhancing the separation of the photogenerated electrons and holes of Pg-C_3_N_4_. Subsequently, the excited electrons reduced oxygen adsorbed on the surface of Pg-C_3_N_4_ to produce superoxide radicals, which reacted with water to achieve hydroxyl radicals. Finally, hydroxyl and superoxide radicals degraded SMX, as shown in eqn (13)–(27).

Production of cavitation bubbles

13 H_2_O + heat (high temp + pressure) → ˙OH + ˙OH (pyrolysis rxn)

14 H_2_O + ˙OH → H_2_ + ˙OH

15 ˙OH + ˙OH → H_2_O_2_

16 O_2_ + ˙H → HO_2_

17 HO_2_ + HO_2_ → O_2_ + H_2_O_2_ → 2˙OH

18 H_2_O_2_ + heat (high temp + pressure) → 2˙OH

19 La_0.8_FO@PgNS + ultrasound → h^+^/VB + e^−^/CB

20 La_0.8_FO@PgNS + hv → La_0.8_FO (e^−^/h^+^)/g-C_3_N_4_ (e^−^/h^+^)

21 La_0.8_FO@PgNS + e^−^ → La^3+^ + O_2_^−^

22 e^−^ (g-C_3_N_4_) + 2H^+^ → H_2_

23 e^−^ (g-C_3_N_4_) + O_2_ → ˙O_2_^−^

24 La_0._8FO (h^+^) + OH^−^/H_2_O → ˙OH

25 La_0._8FO (h^+^) + SMX → degraded products

26 ˙O_2_^−^ + SMX → degraded products

27 ˙OH + SMX → degraded products

## 5 Conclusion

In summary, the sonophotocatalyst La_0_._8_FO@PgNS was successfully synthesized by the citric combustion method. Different techniques were used to characterize it. The EDX and XPS surface composition analysis confirmed the presence of La, C, O, N, and Fe.

The degradation of SMX over La_0.8_FO and La_0_._8_FO@PgNS sonophotocatalyst was investigated. The TOC removal, which depicts the level of mineralization, was 72.10% and 99.60%, respectively. Different operational variables were optimized and the excellent sonophotocatalytic degradation of SMX over La_0_._8_FO@PgNS was due to the hydroxyl and superoxide radicals generated due to the ultrasonic wave, leading to the production of cavitation bubbles under high temperature and pressure. The degradation process of SMX follows the pseudo-first-order kinetic model with the rate constant of 0.01494 min^−1^ and it is stable over 8 cycles, making it a sustainable material.

## Data availability

Crystallographic data for g-C_3_N_4_ and LaFeO_3_ has been deposited in the ICSD under PDF 00-087-1526 and PDF 01-089-1268 and can be obtained from https://icsd.products.fiz-karlsruhe.de/

## Conflicts of interest

There are no conflicts to declare.
